# Adenomyoepithelioma of the Breast in the Setting of Prior Contralateral Breast Malignancy

**DOI:** 10.7759/cureus.39189

**Published:** 2023-05-18

**Authors:** Leah C Dauterman, Kristen Lentsch, Betty Fan

**Affiliations:** 1 Medical Education, Indiana University School of Medicine, Indianapolis, USA; 2 Surgical Oncology, Indiana University School of Medicine, Indianapolis, USA

**Keywords:** lumpectomy, benign breast disease, breast cancer, breast, adenomyoepithelioma

## Abstract

An 81-year-old female patient underwent a screening mammogram one year after completing treatment for right-sided estrogen receptor (ER)/progesterone receptor (PR)-negative ductal carcinoma in situ (DCIS). A new 1-cm mass was noted in the contralateral breast. Ultrasound and percutaneous core needle biopsy results were suggestive of an atypical papillary lesion. An excisional biopsy was performed, and the final pathology was consistent with a benign adenomyoepithelioma (AME). Surgical resection was considered her definitive treatment. AME of the breast is a rare clinical entity, with only a handful of case reports and case series available. In this case report, we review common clinical and radiologic presentations, methods of diagnosis, and recommendations for management based on current literature. The presence of an AME in the background of a previous or synchronous breast malignancy occurs in a very small percentage of cases. On review of available literature, we identified other cases with a past or current history of breast malignancy.

## Introduction

Adenomyoepithelioma (AME) of the breast is a rare tumor that is characterized by biphasic proliferation of both epithelial and myoepithelial cells [1]. Although AME is most often a benign tumor, malignant degeneration has been known to occur and it is considered to be a tumor with a low malignant potential [2]. Initial malignant transformation can take place in one or both of the cell components, with epithelial cell transformation being more common [1]. Due to the exceedingly rare nature of AME, most relevant existing literature consists of only case reports.

AMEs tend to present in patients over 50 years of age but have been known to occur in younger populations [3]. Initial clinical presentation typically involves a single palpable nodule. Diagnosis is challenging as clinical and radiographic findings can be highly variable. Although pathology is the most accurate method of diagnosis, some studies have reported that as many as half of AMEs are later reclassified as another diagnosis after a review of the pathology [4]. We present a rarely encountered case of adenomyoepithelioma found in a patient who had previously been treated for an estrogen receptor (ER)/progesterone receptor (PR)-negative ductal carcinoma in situ (DCIS) in the contralateral breast. This case illustrates the radiologic characteristics of AME along with the typical course of diagnosis and management.

## Case presentation

**Figure 1:**
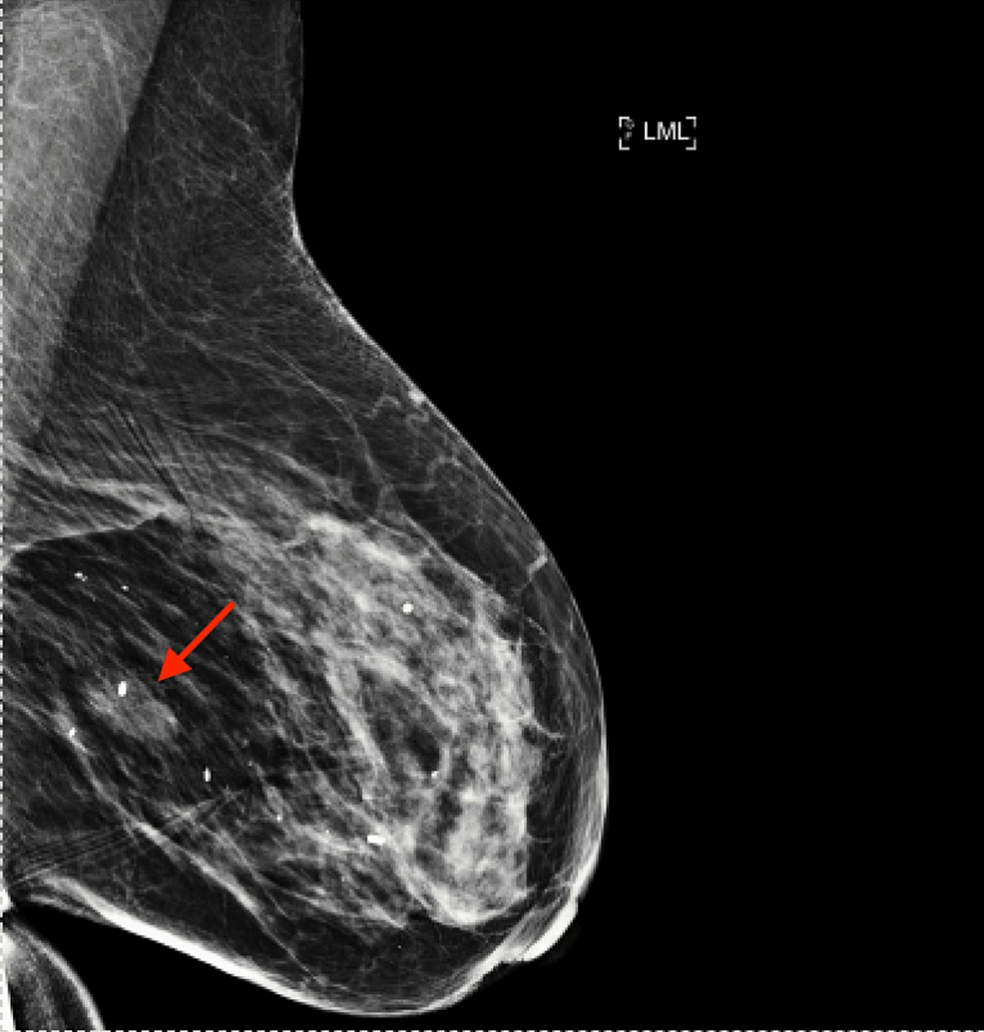
Mammogram with a mediolateral view (LML) that shows the 1-cm oval mass with indistinct margins located at the junction of the middle and posterior third inner central left breast, denoted by arrow. LML: left mediolateral.

The patient is an 81-year-old woman with a history of right-sided ER/PR-negative DCIS treated with lumpectomy and radiation one year prior who was found to have a new left-sided breast mass on screening mammography. Her diagnostic mammogram showed a 1-cm oval mass with indistinct margins in the central area of the left breast (Figure 1). Physical examination showed no dominant masses, lesions, discharge, or skin changes. The patient reported no symptoms.

**Figure 2:**
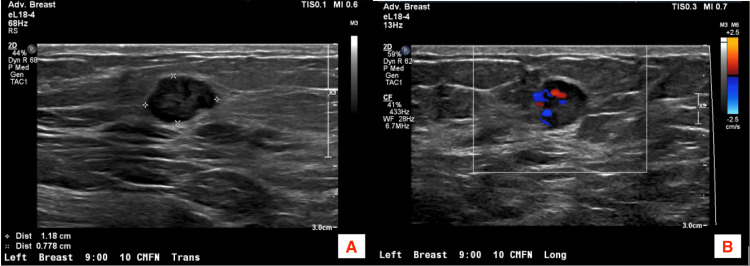
Ultrasound confirms the findings of the mammogram. (A) An ovoid circumscribed hypoechoic mass measuring 1.2 x 0.8 x 0.8 cm at the 9:00 position located 10 centimeters from the nipple (CFN). (B) There is internal vascular flow.

Ultrasound was performed for further evaluation and showed an ovoid circumscribed 1.2 x 0.8 x 0.8 cm hypoechoic mass with indistinct margins at the 9:00 position located 10 cm from the nipple in the left breast. The internal vascular flow was noted. Limited visualization of the left axilla showed morphologically benign lymph nodes (Figure 2).

Ultrasound-guided core needle biopsy was performed two weeks later. Pathology showed a focally monotonous papillary lesion consistent with an atypical papillary lesion. The specimen was negative for malignancy. The excisional biopsy of this mass was recommended for definitive diagnosis due to the atypia noted on core biopsy sampling. The patient underwent a localized excisional biopsy of the left breast. The specimen consisted of a gray-yellow firm granular mass with dimensions measuring 1.6 x 1.2 x 1 cm.

Histopathological analysis of the surgical pathology showed adenomyoepithelioma without evidence of malignancy and negative margins greater than 1 cm from the mass. The patient was seen by the breast surgery clinic one month after the procedure for follow-up. She continues to do well with return to daily activities and reports no new health concerns. Given her good health status and recent abnormal breast findings, she elected to continue regular screening mammograms at this time and is scheduled for a bilateral diagnostic mammogram next year.

## Discussion

AME of the breast is a rare neoplasm that was first described in 1970 by Hamperl et al. [5] and later extensively classified in 1991 by Tavassoli [6]. It is characterized by the proliferation of both epithelial and myoepithelial cells and is therefore classified as a biphasic neoplasm [1]. Diagnosis is often challenging given that clinical and radiologic presentations have been yet to be consistently described. Therefore, diagnosis requires pathology using percutaneous core biopsy for initial diagnosis and excisional biopsy for definitive diagnosis [3,4,7]. Immunohistochemistry is used to demonstrate biphasic proliferation of the surgical specimen [7].

Variable clinical and radiological presentations represent significant challenges in diagnosis. Current literature suggests that some patterns are more common. AMEs are most likely to occur in the fifth to sixth decades of life [8]. These tumors are often solitary, well-circumscribed lesions that are greater than 1 cm in diameter. Clinical presentations can include a palpable mass, enlarging lump, or focal pain with palpable abnormality being the most commonly reported presenting symptom [4]. Existing literature reports the average size of masses as ranging from 2.0 to 2.5 cm, although one case series reports sizes ranging from 0.7 to 2.6 cm [1,3].

Radiologic presentations also tend to be widely varied. Because mammography and ultrasound are commonly used for first-line identification of breast masses, findings from these imaging modalities are the most well-studied. On mammography, findings are non-specific and can include masses, microcalcifications, and focal asymmetry [1]. Lesions are most often described as well-circumscribed, equal density, and oval shaped [9]. Masses are commonly oval or irregular in shape and are hypoechoic (but have also been reported to be heterogeneously hypoechoic) [1-3]. Margins usually are circumscribed or microlobulated. Increased vascularity may be observed. Malignant AMEs have been noted to have more heterogenicity and ill-defined borders [2,9].

Because clinical and radiographic findings are non-specific, differentiation of AMEs from other breast masses is difficult. Current literature notes that the most commonly observed features of AMEs include oval masses or irregular hypoechoic masses with circumscribed or microlobulated margins [10]. Oval masses with circumscribed or microlobulated margins are also commonly seen on ultrasound in fibroadenomas and triple-negative invasive ductal carcinomas. Irregular hypoechoic masses are commonly used to describe invasive breast malignancies [1].

Although the vast majority of patients with AME have no associated malignancy, both ipsilateral and contralateral AMEs have been described in patients with past or concurrent malignancies [1,10]. Interestingly, two of the 27 AMEs originally studied by Tavassoli were found to have concurrent epithelial malignancies within the described AME [6]. Parikh et al. [1] describe an 82-year-old woman with a distant history of breast cancer (type unspecified) in the right breast who presented with a palpable subareolar lump in the left breast found to be a benign AME on pathology. Similarly, Moritz et al. [9] published a case series including a 65-year-old female previously treated for invasive ductal adenocarcinoma with findings of an irregular, ill-circumscribed, and hypoechoic mass on ultrasound, later diagnosed as AME. Other case reports have described concurrent DCIS or invasive ductal carcinoma with AME [4,11-13]. Malignant AME has also been described in the setting of a synchronous phyllodes tumor [9,14]. Overall, AMEs in the setting of prior malignancy in the contralateral breast as seen in the patient described in this case report have not been as frequently observed. While no clear relationship has been established between AME and breast cancers, it is interesting to note that multiple publications noted a higher number of cases with previous or simultaneous malignancy than would be expected. This has prompted clinicians to ponder a possible correlation and whether AMEs confer an underlying increased propensity for other de novo breast cancers to develop [9]. However, a definitive link has yet to be identified, and it is unclear if this is simply a coincidence due to the prevalence of breast cancer in females.

The treatment of AME is debated among clinicians, and guidelines have yet to be established owing to the lack of prospective studies. Based on the current literature, complete surgical resection tends to be sufficient for benign AME, while malignant AME could require additional treatment with radiation or chemotherapy [3]. Clinicians agree that resection should be done with wide excision to reduce the risk of recurrence [7].

## Conclusions

Adenomyoepithelioma of the breast is a rare tumor that is most commonly benign but can undergo malignant transformation. Diagnosis of AME is challenging given its variability in clinical and radiologic presentation. Therefore, tissue pathology is required to make a diagnosis. Guidelines for management have not yet been established as the rarity of AME makes prospective studies difficult. Most clinicians recommend wide surgical excision for definitive diagnosis and treatment.

## References

[1] Parikh P, Jameel Z, Falcon S (2021). Adenomyoepithelioma of the breast: Case series and literature review. Clin Imaging.

[2] Chen F, Feng H, Wu H, Zhong J, Weng X (2022). Malignant adenomyoepithelioma of the breast with contralateral apocrine ductal carcinoma in situ: A rare case. J Clin Ultrasound. Published Online First: 3 October.

[3] Haque W, Verma V, Suzanne Klimberg V (2020). Clinical presentation, national practice patterns, and outcomes of breast adenomyoepithelioma. The Breast Journal.

[4] Wiens N, Hoffman DI, Huang CY, Nayak A, Tchou J (2020). Clinical characteristics and outcomes of benign, atypical, and malignant breast adenomyoepithelioma: a single institution’s experience. Am J Surg.

[5] Hamperl H, Altmann H-W, Benirschke K (1970). Current Topics in Pathology. Springer: Berlin, Heidelberg.

[6] Tavassoli FA (1991). Myoepithelial lesions of the breast. Myoepitheliosis, adenomyoepithelioma, and myoepithelial carcinoma. Am J Surg Pathol.

[7] AlQurashi M, Abdel Hadi M, Binammar AA, Al Muhanna A, Kussaibi H, Al Shammary E (2022). Adenomyoepithelioma of the Breast: A Report of 3 Cases. Am J Case Rep.

[8] Al Mulla L, Abdelhadi M, Al Muhanna A, Elsharkawy T, Al Nemer A (2022). Adenomyoepithelioma of the breast with unusual confounding diagnostic feature: a case report. J Med Case Rep.

[9] Moritz AW, Wiedenhoefer JF, Profit AP, Jagirdar J (2016). Breast adenomyoepithelioma and adenomyoepithelioma with carcinoma (malignant adenomyoepithelioma) with associated breast malignancies: A case series emphasizing histologic, radiologic, and clinical correlation. Breast.

[10] Uchida M, Gatica C, Hasson D, Gallegos M, Pinochet MÁ (2021). Breast adenomyoepithelioma from a radiologic perspective. Radiologia (Engl Ed.

[11] Kuroda N, Fujishima N, Ohara M, Hirouchi T, Mizuno K, Hayashi Y, Lee G-H (2008). Coexistent adenomyoepithelioma and invasive ductal carcinoma of the breast: presentation as separate tumors. Med Mol Morphol.

[12] Kamei M, Daa T, Miyawaki M, Suehiro S, Sugio K (2015). Adenomyoepithelioma of the breast coexisting with ductal carcinoma in situ: a case report and review of the literature. Surg Case Rep.

[13] Kwon SY, Bae YK, Cho J, Kang SH (2011). Myoepithelial carcinoma with contralateral invasive micropapillary carcinoma of the breast. J Korean Surg Soc.

[14] Buch A, Rout P, Makhija P (2006). Adenomyoepithelioma with phyllodes tumor--a rare combination in a solitary breast lump. Indian J Pathol Microbiol.

